# Recommendations for Lifestyle Physical Activity and Exercise During the Perinatal Period: A Narrative Review

**DOI:** 10.3390/healthcare14010122

**Published:** 2026-01-04

**Authors:** Józef Opara, Krzysztof Mehlich, Jarosław Szczygieł

**Affiliations:** 1 Institute of Physiotherapy and Health Sciences, Academy of Physical Education, ul. Mikołowska 72b, 40-065 Katowice, Poland; k.mehlich@awf.katowice.pl (K.M.); j.w.szczygiel@awf.katowice.pl (J.S.); 2 Department of Neurology, Clinical Hospital No.1 Named After Prof. Stanisław Szyszko, Silesian University of Medicine, ul. 3 Maja 13/15, 41-800 Zabrze, Poland

**Keywords:** lifestyle physical activity, physical activity, pregnancy, perinatal period, postpartum, recommendations

## Abstract

Background: The World Health Organization (WHO) has recently focused much attention on physical activity recommendations. Regular physical activity offers broad health benefits, reducing the risk of some chronic diseases and improving bone structure and muscle strength. Although the scientific literature provides numerous recommendations for physical activity during pregnancy and the postpartum period, there are no official recommendations for lifestyle-related physical activity. Objectives: This narrative review aimed to review the current knowledge on physical activity during pregnancy and the postpartum period, specifically focusing on lifestyle-related physical activity. The review was based on the definition of lifestyle-related physical activity proposed by Dunn et al. in 1998, which is at least 30 min of self-selected activity per day, encompassing all recreational, occupational, or household activities, as well as planned and unplanned activities that are part of daily life. Methods: A number of databases were analyzed, including PubMed, the Cochrane Library, Embase, and Web of Science. Results: The most valuable reports and recommendations regarding physical activity during the perinatal period were identified. Conclusions: Moderate physical activity during pregnancy is safe and offers benefits, such as reducing the risk of gestational diabetes, preeclampsia, and excessive weight gain, as well as improving mental health. The most common benefits of continuing physical activity after delivery include weight control, reduced risk of depression, and improved quality of life. Lifestyle-based physical activity is easier to implement and more achievable than structured exercise. Further research is needed to establish recommendations regarding lifestyle-based physical activity during the perinatal period.

## 1. Introduction

This narrative review of the literature aimed to summarize the existing knowledge on the role of PA and related recommendations during pregnancy and postpartum, i.e., during the perinatal period.

In recent years, one can observe the WHO’s growing interest in promoting physical activity (PA) [1]; several similar scientific articles have been published. In 2020, in a special issue of the British Journal of Sports Medicine, Bull et al., on behalf of WHO experts, presented recommendations for adults to engage in 150–300 min of moderate-intensity exercise or 75–150 min of vigorous-intensity exercise per week, or an equivalent combination of both. The experts noted that regular muscle-strengthening exercise plus reduced sedentary behavior provides health benefits for people of all ages and in all situations. However, there was no precise definition of what constitutes a sedentary lifestyle [2].

PA is promoted in virtually all fields of medicine as a primary prevention strategy and to reduce the various adverse effects of a sedentary lifestyle. The term “PA” encompasses both intentional movement (e.g., exercise) and unintentional movement (e.g., daily activities). Numerous studies have confirmed that physically active individuals have a lower risk of premature morbidity and mortality from many chronic conditions [3,4]. The WHO still uses the old definition of physical activity developed by Caspersen et al. in 1985, who defined it as “any bodily movement generated by skeletal muscles that results in energy expenditure.” The authors classified exercise as a subset of physical activity, consisting of planned, structured, and repetitive activities with the ultimate or intermediate goal of improving or maintaining physical fitness [5].

The key to our review was the definition of lifestyle physical activity, created in 1998 by Dunn et al., which states: “Lifestyle physical activity is a daily accumulation of at least 30 min of self-selected activity, which includes all recreational, occupational, or household activities of at least moderate to vigorous intensity; these may be planned or unplanned activities that are part of daily life” [6].

Lifestyle PA is a bodily movement that results in energy expenditure as part of daily life, rather than through structured exercise. It can be planned or unplanned; it includes activities such as shopping, gardening, housework, climbing stairs instead of the elevator, walking or cycling to work, and taking walks during work breaks. It can also include recreational activities such as dancing, light walking, or even gaming. This activity is spread throughout the day and integrated into daily life, so movement becomes a routine. It is worth adding here that most authors, when describing the PA lifestyle, forget about childcare, which is also related to physical effort.

Low PA among young women and a high prevalence of obesity and cardiometabolic diseases are common. This makes increasing PA among women of reproductive age before, during, and after pregnancy a major public health challenge. Fell et al. (2009) [7] compared the physical activity levels of 1737 previously active American women during the first 20 weeks with their activity levels in the year before pregnancy. The greatest decline in activity was observed in sports and recreation, with a smaller decline in household activities. Factors such as age under 35, having many children, lower education, being overweight, and low levels of PA before pregnancy adversely affected activity levels in early pregnancy [7].

Based on the results of a survey conducted among 110 women in their third trimester of pregnancy, Torbé et al. (2013) [8] stated that women’s knowledge of the need for and opportunities for PA during pregnancy is unsatisfactory. This level of knowledge was particularly low among women under 25, primiparas, unmarried women, and those with only primary education [8]. Nascimento et al. (2015) [9] recruited 1279 women within 72 h of delivery in a cross-sectional study in Campinas, São Paulo, southeastern Brazil. The prevalence of PA among participants was lower during pregnancy than before. Half of the women discontinued PA due to pregnancy. The lowest proportion of women engaging in PA was observed in the first and third trimesters of pregnancy. The most frequently reported type of exercise was walking, followed by aqua aerobics. Factors positively associated with exercise included higher education level, priority at delivery, exercise before pregnancy, and exercise guidance during prenatal care. Pregnant women most frequently reported moderate-intensity exercise and housework [9]. In 2016, Hesketh and Evenson published the results of a review of the 2007–2014 National Health and Nutrition Examination Survey (NHANES). They interviewed 247 pregnant women aged 20–44, with a mean age of 28.8 (SD = 6.0) and a mean BMI of 29.6 (SD = 8.0); 65% of the women had a college degree. They found that 60% of the women surveyed reported not engaging in any form of recreational activity (Leisure Time PA) [10].

## 2. Benefits of Physical Activity During Pregnancy

According to Gascoigne et al., approximately 48% of pregnant women in the United States gain more weight than recommended. Excessive weight gain during pregnancy is associated with an increased risk of maternal and fetal complications, including preterm birth, preeclampsia, and gestational diabetes, which, consequently, results in more serious adverse health outcomes in the short and long terms for both mothers and their offspring [11]. Many studies have shown that patients whose pregnancies are complicated by gestational diabetes and preeclampsia have an increased risk of almost all subtypes of cardiovascular diseases, including stroke, myocardial infarction, thromboembolism, and death, compared with women without pregnancy complications [12,13,14,15].

In 2019, DiPietro et al., acting on behalf of the members of the Physical Activity Guidelines Advisory Committee, published the results of an umbrella review of the benefits of PA during pregnancy and postpartum. A total of 76 systematic reviews and meta-analyses from 2006 to 2018 met the criteria. They found strong evidence that moderate-intensity PA reduces the risk of excessive gestational weight gain, gestational diabetes, and postpartum depressive symptoms. Limited evidence suggested an inverse association between PA and the risk of preeclampsia, gestational hypertension, antenatal anxiety, and depressive symptoms. There was insufficient evidence for the effect of PA on postpartum weight loss, postpartum anxiety, and impact during and after pregnancy. It was unclear whether these associations differed by age, race, socioeconomic status, or pre-pregnancy weight [16]. Cannon et al. (2023) [17] performed a systematic review on the association between PA and sleep during pregnancy. Poor sleep during pregnancy had negative effects on maternal and child health. Ten studies were included in the review. Five of the ten studies used observational data collection methods, and the remaining five used interventional methods. Eight of the ten included studies found that PA (including exercise) was positively associated with sleep quality during pregnancy. Therefore, PA may be an effective non-pharmacological strategy for improving sleep during pregnancy [17].

## 3. History of Recommendations on Physical Activity in Pregnancy

Decades ago, grandmothers advised their pregnant granddaughters to eat plenty, rest, and gain weight. In the 21st century, health-promoting behaviors, including various forms of PA, are increasingly common. It is now common knowledge that PA is essential to prepare for childbirth. On the other hand, the percentage of pregnancies with various complications has increased. In recent years, more and more recommendations regarding PA in uncomplicated pregnancies have emerged.

Burnett states that the authors of the Bible had already noticed that the births of Hebrew slaves were easier than those of their settled Egyptian mistresses [18]. Also, Artal et al. mentioned that both Aristotle (4th Century B.C.E.) and later Plutarch of Chaeronea (1st/2nd Century C.E.) urged Spartan and other Greek women to exercise to decrease the pain of childbearing [19]. As recently as the late 19th century, pregnant women were encouraged to stay at home, maintain “isolation”, and avoid straddling exercises during gymnastics to prevent the risk that female organs “might slip” [20].

The 20th century saw a radical shift in the approach to PA in general, and PA during pregnancy in particular. In the 1930 s, prenatal exercise classes led by physiotherapist Heardman were introduced in England and Sweden [21]. In 1959, Karmel and Bing popularized the still-popular Lamaze method in the United States, which encompasses physiological and psychological preparation for childbirth [22].

## 4. Perinatal Physical Activity

### 4.1. Methods of Narrative Review

We aimed to identify all available English-language randomized controlled trials (RCTs) that evaluated the perinatal PA, especially lifestyle PA. We defined the perinatal period as the time between one year before conception and one year after birth.

A search was performed in PubMed, EBSCO, Embase, Cochrane, Web of Science, and Science Direct databases, with searches conducted from 1980 to 31 October 2025. We used medical subject headings (MeSH) and full texts related to pregnancy, physical activity, lifestyle physical activity, perinatal period, and post-partum. Exclusion criteria were (1) studies involving participants with female-related problems other than pregnancy; (2) studies with incomplete data or unable to obtain statistical analysis; (3) studies on qualified sports and intensive PA; (4) studies without control groups or involving only single protocols, abstracts, or conference poster presentations; (5) studies with a non-randomized controlled design.

### 4.2. Results

A total of 560 articles were found, and of those, 31 were suitable for meta-analysis. There were many reports on the PA in pregnancy and post-partum, but there were relatively few papers on perinatal lifestyle PA. Different guidelines exist for previously inactive women and previously active women.

Numerous guidelines and definitions of PA have been used to study PA during pregnancy. The American College of Obstetricians and Gynecologists (ACOG) has been the most active. The first guidelines were published in 1985, with subsequent ACOG recommendations published in 1994, 2002, 2015, and 2020 [23,24,25,26,27,28]. The 1985 ACOG Technical Bulletin, “Exercise in Pregnancy and the Postpartum Period,” was the first comprehensive exercise recommendation for pregnant women, establishing guidelines for physical activity during pregnancy. It set a heart rate limit of 140 beats per minute for women exercising during pregnancy [24]. Subsequent ACOG recommendations removed this absolute heart rate limit and focused on individual exercise programs during pregnancy, encouraging pregnant women to maintain exercise intensity “from relatively light to slightly vigorous.”

ACOG Committee members’ opinion No. 650 from 2015 became well known. It has been cited by many authors and can be considered a milestone in the development of recommendations for PA in pregnancy and the postpartum period; it inspired other countries to create their own recommendations. ACOG experts concluded that regular exercise provides significant health benefits for pregnant women and should be promoted by physicians. The authors noted at the beginning that PA at all stages of life maintains and improves cardiovascular and respiratory fitness, reduces the risk of obesity and related comorbidities, and prolongs life. PA during pregnancy is associated with a reduced risk of obesity and its consequences. Women with uncomplicated pregnancies should be encouraged to engage in aerobic and conditioning exercise before, during, and after delivery. Regular PA during pregnancy improves or maintains physical fitness, helps with weight management, reduces the risk of gestational diabetes in obese women, and improves psychological well-being. An exercise program that ultimately achieves the goal, including at least 20–30 min of moderate-intensity exercise per day on most or all days of the week, should be developed collaboratively with the patient and modified as medically indicated. Experts also listed examples of safe and unsafe forms of exercise for pregnant women. Safe activities include walking, Nordic walking, swimming, stationary cycling, and jogging (for women who practiced these activities before pregnancy). Unsafe activities include contact sports (ice hockey, boxing, soccer, and basketball) and activities associated with a high risk of falling (e.g., horseback riding, apparatus gymnastics, water skiing, surfing, and diving). It was concluded that women who were not active before pregnancy should gradually increase their exercise, while those who exercised intensively before pregnancy may continue their activity during pregnancy. It was also recommended to continue exercising after delivery, depending on their well-being and health. Before recommending an individual exercise program, the physician should thoroughly assess the health of the pregnant woman and identify PA limitations and contraindications that pose a risk to the health of the mother and her child [27].

The 2015 ACOG document was updated five years later to define the latest evidence regarding the benefits and risks of PA and physical exercise during pregnancy and postpartum. Experts confirmed that PA, consisting of planned, structured, and repetitive body movements aimed at improving one or more components of physical fitness, is an important component of a healthy lifestyle, and obstetricians-gynecologists and other healthcare providers should encourage their patients to continue or initiate exercise. Women who regularly participated in vigorous aerobic exercise or were physically active before pregnancy can continue this activity during pregnancy and postpartum. Observational studies of women who exercised during pregnancy have shown a reduced risk of gestational diabetes, cesarean section, and shorter postpartum recovery. PA has also been shown to be an important factor in preventing depressive disorders in women in the postpartum period [28].

In a search of Medline and PubMed up to 2012, Evenson et al. found 11 articles from around the world, originating from specific countries (Australia, Denmark, France, Spain, Japan, Canada, the United Kingdom, and the United States). Data related to the recommendation to mandate the use of PA during pregnancy (10/11) and included information on its frequency (9/11) and duration (9/11). The regulation mandated exercise during pregnancy (10/11). Six documents contained absolute and relative contraindications to exercise [29]. In a broad commentary, the authors noted that the content of the guidelines varied depending on publication date and target audience (i.e., clinicians vs. public health workers). Almost all guidelines recommended moderate-intensity physical activity during pregnancy, and most also suggested that women consult a physician before starting or continuing an exercise program. All excluded sports were associated with a risk of falls, injuries, or collisions during pregnancy. The difference between the guidelines was the use of relative and absolute intensity to determine exercise intensity. Canada, Japan, and the United Kingdom provided intensity guidance using both heart rate (absolute intensity) and ratings of perceived exertion (relative intensity). While most guidelines focused on aerobic exercise, five countries recommended strengthening exercises. Only a few studies have examined the safety and effectiveness of strength training during pregnancy. Given the potential health benefits of strength training during pregnancy, further research is needed to determine the safest recommendation in terms of volume and intensity. Canadian guidelines suggest using lighter weights and higher repetitions, avoiding lifting weights in the supine position, and avoiding the Valsalva maneuver. A challenge in several guidelines has been the lack of clarity in the terminology describing exercise. For example, in several guidelines (Denmark, Norway, the United Kingdom, ACOG USA), the term “exercise” has been replaced by “physical activity.” Some terminology issues stem from cultural differences. For example, the Danish guidelines use the terms “physical activity” and “exercise” interchangeably, and also use other terms (physical training, movement, fitness training) that are difficult to translate directly. Despite differences in the recommended amount of PA during pregnancy, pregnant women worldwide often fail to meet these recommendations. Studies conducted in each country included in this review showed that many pregnant women do not engage in the amount of PA or exercise recommended in their country. Promoting PA during pregnancy, including both education and addressing barriers to physical activity, should be prioritized. Future research in different countries could clarify the reasons for differences in beliefs, investigate whether cultural factors contribute to these differences, and determine whether tailored messages are more effective than generic educational approaches. To facilitate the practical application of the guidelines, a Prenatal PA Readiness Assessment (PAR) tool was developed to facilitate communication between the healthcare professional, fitness specialist, and pregnant woman. The four-page form includes a pre-exercise checklist for the woman to complete, contraindications to exercise for the healthcare professional to complete, and a health assessment form for the prenatal fitness specialist to use. It also includes information on recommendations for aerobic activity, muscle conditioning, and an active lifestyle [29,30].

Canadian guidelines for PA were published in 2019 by Mottola et al. Based on a literature review, experts drew the following conclusions: 1. All women without contraindications should be physically active during pregnancy. 2. Pregnant women should engage in at least 150 min of moderate-intensity physical activity per week to achieve clinically significant health benefits and reduce the risk of pregnancy complications. 3. PA should be undertaken at least three times per week; however, daily activity is recommended. 4. To maximize benefits, pregnant women should incorporate a variety of aerobic exercises and resistance training into their training. Adding yoga and/or gentle stretching may also be beneficial. 5. To reduce the risk of urinary incontinence, pelvic floor muscle exercises (PFMT) (e.g., Kegel exercises) can be performed daily. 6. Pregnant women who experience dizziness, nausea, or discomfort while exercising in a supine position should modify their exercise position. The experts also added that pregnant women should avoid PA in excessive heat, especially when humidity is high. They should also avoid diving. Women living below 2500 m above sea level should avoid PA at high altitudes (>2500 m) [31].

The WHO, in its 2020 guidelines on PA and sedentary behavior, in the chapter on women during pregnancy and after childbirth, stated that PA during pregnancy and after childbirth has benefits for maternal and fetal health: it reduces the risk of preeclampsia, gestational hypertension, gestational diabetes, excessive gestational weight gain, perinatal complications and postnatal depression, and also reduces neonatal complications; it has no negative impact on birth weight or an increased risk of stillbirth. It is recommended that all pregnant and postpartum women who have no contraindications engage in regular PA during pregnancy and postpartum (strong recommendation, moderate-certainty evidence). To obtain significant health benefits, at least 150 min of moderate-intensity aerobic PA per week should be achieved (strong recommendation, moderate-certainty evidence). There is high-certainty evidence that PA during pregnancy is associated with reduced gestational weight gain and a reduced risk of gestational diabetes in overweight or obese pregnant women. There is moderate-certainty evidence of a small but significant reduction in the risk of preterm birth in mothers who engage in vigorous PA. PA should be gradually resumed after delivery, and in the case of a cesarean delivery, only after consultation with a physician [32].

Australian guidelines for PA during pregnancy and postpartum were published in 2022. Brown et al. conducted a review of the scientific evidence and concluded that PA and/or exercise during pregnancy and postpartum are safe, and they provide health benefits to the woman and her unborn child and may reduce the risk of some pregnancy complications. Four specific guidelines addressed the modification of PA to accommodate the physical changes that occur during pregnancy; for instance, performing pelvic floor muscle exercises during and after pregnancy has been recommended. Warning signs and contraindications to PA and/or exercise during pregnancy were also identified [33].

Budlers (2022) [34], performing a systematic review, found 20 articles in which the number of participants varied between 20 and 1023 pregnant women. They stated that pregnant women receive questionable information about PA during pregnancy in internet. Aerobic exercise, lumbar stabilization and stretching exercises, aquatic exercise, nerve and tendon gliding exercises, resistance training, and strength training have been reported to benefit the health and well-being of pregnant women. For all types of exercise, moderate-intensity exercise is recommended throughout pregnancy [34].

As a result of a scoping review of public health guidelines for physical activity during pregnancy from around the world published since 2010, Hayman et al. (2023) [23] identified 30 guidelines, published in 11 different languages, developed or endorsed by government agencies. For women with uncomplicated pregnancies, guidelines most often recommend 150–300 min per week of moderate-intensity aerobic activity; pelvic floor muscle strengthening exercises; and modifications to some exercises (e.g., lying down). These guidelines include a list of warning signs for cessation of PA (e.g., persistent dizziness, vaginal bleeding) and activities to avoid (e.g., those at high risk of falling or collision). Few guidelines provide specific advice for women with high PA (e.g., athletes) or address specific trimesters or cultural factors [23].

In 2024, the Polish Society of Gynecologists and Obstetricians (PTGiP), in conjunction with the Polish Society of Sports Medicine (PTMS), published recommendations regarding PA during pregnancy and the postpartum period. Polish recommendations are no longer as conservative as before. It has been emphasized that PA during pregnancy benefits both the mother and the developing fetus. In the absence of medical or obstetric contraindications, physical activity during pregnancy carries no risk and is recommended for regular exercise throughout pregnancy. Modifications to the exercise plan may be necessary to accommodate changes in the woman’s body during pregnancy and the needs of the fetus. PA is also crucial for a woman’s health after childbirth. It has also been emphasized that it is necessary to motivate women to continue or initiate PA during pregnancy and after childbirth. This is the responsibility of physicians, midwives, trainers, and physiotherapists. Collaboration with qualified instructors (personal coaches), trainers, and physiotherapists is recommended [35].

Evenson et al. (2024) [36] published a review of public health guidelines for postpartum (six to 12 weeks after delivery) PA and sedentary behavior from around the world. A systematic database search since 2010 identified 22 countries with guidelines on PA and sedentary behavior in the postpartum period and 11 countries with guidelines on sedentary behavior. Guidelines were developed for specific continents: Europe (*n* = 12), Asia (*n* = 5), Oceania (*n* = 2), Africa (*n* = 1), North America (*n* = 1), and South America (*n* = 1). The most frequently mentioned benefits of PA were weight control (*n* = 10), reduced risk of postpartum depression or depressive symptoms (*n* = 9), and improved mood/well-being (*n* = 8). Regarding postpartum activity, the most frequently recommended activities were pelvic floor muscle exercises (*n* = 17); muscle strengthening, strength training, or resistance exercises (*n* = 13); aerobics or general aerobic activity (*n* = 13); walking (*n* = 11); cycling (*n* = 9); and swimming (*n* = 9). Limiting prolonged sitting and interrupting sitting for PA were recommended [36].

Recently (2025), Worska et al. discussed the contradictions and convergences in recommendations for PA in pregnancy across countries following the publication of the WHO guidelines in 2020. After analyzing 10 eligible guidelines published in English by eight countries and two international organizations since 2020, it was found that all recommended moderate-intensity PA during pregnancy. Seven documents also recommended vigorous-intensity or high-intensity PA. Some guidelines have recommended PA only after consultation with a physician. The guidelines converged in terms of PA frequency and duration, suggesting at least 150 min per week [37].

In 2015, Muktabhant et al. published a Cochrane Systematic Review on the prevention of excessive weight gain in pregnancy (diet, exercise, or both?). The review included a total of 65 RCTs, 49 of which, involving 11,444 women, provided data for the quantitative meta-analysis. Interventions included diet alone, exercise alone, or a combination of diet and exercise, usually compared with standard care. Diet, exercise alone, or both reduced the risk of excessive weight gain (GWG) by an average of 20%. Interventions including low-glycemic index diets alone, supervised or unsupervised exercise, or both diet and exercise led to a similar reduction in the number of women with GWG. These studies also demonstrated a reduction in the risk of GWG. Other benefits included a lower risk of cesarean delivery, macrosomia, and neonatal respiratory disease, particularly in high-risk women receiving interventions combined with diet and exercise. Blood pressure in mothers with hypertension may also be reduced [38]. In the same year, Davenport et al. published the results of a systematic review and meta-analysis of 52 reports (*n* = 131,406) on the effects of prenatal exercise on symptoms of anxiety and depression in both the prenatal and postnatal periods. “Moderate”-quality evidence from randomized controlled trials (RCTs) showed that exercise-only interventions reduced prenatal depressive symptoms and the risk of prenatal depression compared with no exercise [39].

Recently (2025), Zielke et al. stated that regular PA is important for maintaining health throughout life, and especially during the perinatal period. Based on a review of US guidelines, they concluded that at least 150 min of moderate-intensity PA per week is recommended for all three stages of the perinatal period: women trying to conceive, pregnant women, and women for one year after giving birth [40].

## 5. Precautions, Limitations, and Contraindications to Perinatal Physical Activity

Precautions, limitations, and contraindications to perinatal PA are listed in some national guidelines. They differ depending on the PA of women preceding pregnancy, dividing them into physically active and inactive. All authors agree that the type of PA and the level of exercise load should be consulted with a doctor and a physiotherapist. Among unsafe activities, contact sports (ice hockey, boxing, soccer, and basketball) and activities associated with a high risk of falling (e.g., horseback riding, apparatus gymnastics, water skiing, surfing, and diving) have been listed [29]. As for warning signs for cessation of PA, persistent dizziness and vaginal bleeding have been identified [23].

Meah et al. searched databases and identified 44 studies presenting data on pregnant women with contraindications to exercise. Contraindications were categorized as absolute and relative. Their conclusions were that most conditions listed as contraindications were based on expert opinion; there is little empirical evidence supporting the harm of exercise and the benefits of activity restriction. They identified 11 complications (e.g., gestational hypertension, twin pregnancy) previously classified as contraindications for which women may benefit from regular perinatal PA, with or without modifications. Evidence suggests that severe cardiovascular and respiratory disease, placental abruption, vasa previa, uncontrolled type 1 diabetes, intrauterine growth restriction, active preterm labor, severe preeclampsia, and cervical insufficiency are associated with a high potential for maternal/fetal harm and justify classification as absolute contraindications to exercise. The authors believe that removing barriers to PA during pregnancy in women with certain medical conditions may actually benefit maternal and fetal health [41].

## 6. Discussion

Parker et al. stated that women of childbearing age are defined as women ranging from 16 years to 49 years [42]. Helfer stated that the perinatal period should span from the year before the birth of a child to 18–24 months after [43]. Therefore, considerations of PA during pregnancy and the postpartum period should also include the period before conception. When planning motherhood, a modern woman should consider her PA.

The numerous definitions of PA make it difficult to compare different definitions of PA in pregnancy and the postpartum period. A physically active lifestyle takes various forms, depending on the place where it is undertaken (it may be a place of work or home) and the purpose (it may be sport or physical fitness). According to Strath et al. (2013), four dimensions of PA can be listed: 1. method or type of PA, 2. frequency (daily, several times a week), 3. duration (e.g., 15 min, 30 min, 45 min, 60 min, 90 min), and 4. intensity (low, moderate, high) [44]. Fletcher et al. presented the scientific position paper of the American Heart Association in 2013. They defined PA as relatively vigorous (based on maximum heart rate calculated from an age-specific table) and vigorous (based on metabolic equivalent of task (MET). One MET is defined as the amount of O2 a person consumes at rest, calculated as 3.5 mL O_2_/kg body weight/min or 1 kcal/kg/h or 4.184 kJ/kg/h). For example, moderate-intensity exercise involves 50–69% of maximum heart rate, and METs of 3.0–5.9, and vigorous-intensity exercise occurs at 70–89% of maximum heart rate and above METs of 6. In daily life, eating a meal consumes 1–1.5 METs, and taking a shower consumes 3–3.5 METs [45].

Whitaker et al. recently presented the 2025 American Heart Association (AHA) guidelines on sedentary and low-intensity PA during pregnancy and cardiovascular health, issued by the Council on Cardiac Lifestyle and Health, the Council on Cardiac and Stroke Nursing, and the Council on Cardiovascular Surgery and Anesthesiology. Experts highlighted pregnancy complications associated with an increased risk of cardiovascular disease and eight key health factors (including blood pressure, lipids, glucose, and weight gain during pregnancy). Preliminary evidence suggests an association between sedentary behavior and an increased risk of hypertension in pregnancy, shorter gestational age at delivery, low or high fetal birth weight, and increased maternal blood pressure, lipids, glucose, and weight gain during pregnancy. The results regarding low-intensity PA are limited by fewer studies and are less convincing. The benefits of moderate-to-vigorous physical activity (MVPA) during pregnancy for maternal cardiovascular health are well documented. There is strong evidence that MVPA during pregnancy is associated with a lower risk of excessive gestational weight gain and gestational diabetes. Growing evidence also suggests an inverse association between MVPA and the risk of hypertension in pregnancy. Unfortunately, pregnant women face many barriers to MVPA, and the rate of meeting PA guidelines during pregnancy is low [46].

Walking in community (for transportation) is quite a different issue. In 1996, the US Surgeon General recommended at least 30 min brisk walking per day [47]. In 2011, Tudor-Locke et al. recommended between 5200 and 5900 steps per day in two trips [48]. The World Health Organization (WHO) recommends at least 150 min per week of moderate-intensity aerobic PA for adults, e.g., brisk walking for about 30 min per day or walking about 4–5 km most days of the week. For fitness or weight loss, walking 8000 to 10,000 steps (about 6.4 to 8 km) per day is recommended [31].

In 2022, Schantz et al. stated that walking outdoors required approximately 30% more energy per km compared to walking on a horizontal treadmill. They studied middle-aged pedestrians (10 men and 10 women) who regularly commute to work in the Stockholm metropolitan area in a laboratory setting: at rest and at maximum load using a treadmill and a cycle ergometer. Based on their study, the authors recommend 6000 steps per day, or the equivalent for five workdays per year, for optimal health benefits [49].

In a scoping review in 2024, May et al. presented the barriers and facilitators of PA during pregnancy and postpartum in Iranian women. Analysis of 33 studies led the authors to conclude that pregnant and postpartum women in Iran face a variety of barriers to PA. The main barriers were divided into three areas: intrapersonal variables (physical and emotional dimensions), interpersonal factors (cultural poverty, social beliefs, and the role of family members), and environmental factors (organizational structures and economic situation). Pregnancy and the postpartum period were identified as opportunities to educate women about the benefits of PA and a healthy lifestyle. In this review, the authors highlighted facilitators and barriers that are crucial in designing and implementing PA interventions for Iranian pregnant and postpartum women. Preparing this review, the authors found data that, despite guidelines recommending that women without contraindications should engage in PA, fewer than 19% of women achieve the recommended PA levels during pregnancy and postpartum. Finally, the authors emphasized the role of professionals and social support from neighbors, family, and friends to enhance educational efforts and eliminate barriers to PA. Efforts to reduce the lack of PA through education and training of professional staff should be intensified; a diverse and well-trained group of professionals offers a promising opportunity to improve PA levels [50].

Amiri-Farahani et al. developed and validated the psychometric properties of the questionnaire Barriers to Physical Activity during Pregnancy Scale (BPAPS). Exploratory factor analysis resulted in a 29-item scale. These items were then divided into four main factors: intrapersonal barriers related to pregnancy, intrapersonal barriers not related to pregnancy, interpersonal barriers, and environmental barriers. Finally, the internal consistency and stability of the scale were found to be high—Cronbach’s alpha coefficient was 0.824, and test–retest reliability was 0.87. The results indicate that the 29-item scale assessing barriers to PA in pregnant women is a valid and appropriate tool [51].

## 7. Conclusions

Despite advances in modern medicine and promotion by the WHO, most young women do not appreciate the benefits of maintaining PA, even in the 21st century. Meantime, there is strong evidence that PA is safe if performed at moderate and high intensity and in consultation with a personal coach. When planning PA, it is important to consider any contraindications and necessary precautions. The type of PA will depend on the patient’s preferences and capabilities, as well as her physical condition, economic situation, and local conditions. Many experts recommend personalized, daily PA as the first option during pregnancy and postpartum. PA practiced during pregnancy offers several benefits, such as reducing the risk of gestational diabetes, preeclampsia, and excessive weight gain, as well as improving mental health. Postpartum women should aim for at least 150 min of moderate-intensity aerobic activity per week. For a rough estimate of intensity, it is best to use the “conversation test”—you should be able to hold a conversation during exercise.

The most frequently reported benefits of PA are weight control, a reduced risk of postpartum depression or depressive symptoms, lower levels of stress urinary incontinence (SUI), and improved mood or well-being. The most frequently recommended postpartum activities are pelvic floor muscle exercises (PFMEs), muscle strengthening, strength training, resistance exercises, aerobics or general aerobic activity, walking, Nordic walking, cycling, and swimming.

While the literature offers numerous recommendations for PA during pregnancy and the postpartum period, there are no official recommendations for lifestyle PA. Considering the current state of knowledge presented above, 150 min of moderate-intensity lifestyle PA, including brisk walking for approximately 30 min per day or approximately four to five kilometers, can be recommended during the perinatal period. Women with low PA levels who are planning to become mothers should increase their activity levels well in advance. PA can commence 12 h after vaginal delivery and 48 h after cesarean section, provided they feel capable. In the following weeks, exercise intensity should be gradually increased, and a full return to more intense exercise should occur after the postpartum period and after obtaining medical approval.

The practical usefulness of lifestyle PA stems from the fact that it is easier to perform and in some situations more achievable than structured exercise. Further research is needed to establish recommendations for perinatal lifestyle PA.

## Data Availability

No new data were created or analyzed in this study.
